# Enhancing Rare Disease Awareness and Education Among Medical Professionals and Students in Türkiye

**DOI:** 10.1111/jep.70242

**Published:** 2025-08-12

**Authors:** Öznur Karagöz, Buşranur Tırtır, Dilek Güneş, Özge Özgen, Mustafa Özçetin, Gülbin Gökçay, Gülden F. Gökçay, Abdolrahman S. Nateri, Fatmahan Atalar

**Affiliations:** ^1^ Department of Family Health, Child Health Institute Istanbul University Istanbul Türkiye; ^2^ Institute of Graduate Studies in Health Sciences Istanbul University Istanbul Türkiye; ^3^ Rare Diseases Research Laboratory, Istanbul Medical School Istanbul University Istanbul Türkiye; ^4^ Department of Rare Diseases, Child Health Institute Istanbul University Istanbul Turkiye; ^5^ Department of Social Pediatrics, Child Health Institute Istanbul University Istanbul Türkiye; ^6^ Division of Pediatric Nutrition and Metabolism, Istanbul Medical Faculty Istanbul University Istanbul Türkiye; ^7^ School of Medicine, Cancer Genetics & Stem Cell Group, BioDiscovery Institute, Academic Unit of Translational Medical Sciences Nottingham UK

**Keywords:** clinicians, information needs, medical curriculum, medical education, rare diseases, survey

## Abstract

**Purpose:**

Rare diseases (RDs), which are often chronic, degenerative, and life‐threatening conditions, pose significant challenges due to their complexity and limited awareness among healthcare professionals. This study assessed the knowledge, awareness, and educational needs related to RDs among 5th‐ and 6th‐year medical students at Atatürk University, Başkent University, and Istanbul University, as well as pediatric and non‐pediatric specialists in Türkiye, with a focus on differences between these groups.

**Materials and Methods:**

A total of 258 physicians and 132 medical students participated. Data were collected through surveys examining demographics, self‐assessed knowledge, awareness, and perceptions of RD‐related education. Statistical analyses evaluated differences in knowledge and awareness across the groups.

**Results:**

Pediatric specialists reported significantly higher self‐assessed RD knowledge than non‐pediatric specialists. However, both groups showed notable gaps in awareness, particularly concerning RD prevalence and diagnostic timelines in Türkiye. Most participants expressed interst in further education but were unaware of available resources. Among students, 65.9% rated their RD knowledge as ‘Poor’ or ‘Very Poor,’ with no significant differences observed across institutions despite curriculum variations.

**Conclusion:**

The findings highlight a critical lack of competence in RD‐related knowledge among healthcare professionals, especially non‐pediatric specialists. To address this gap, we recommend integrating integrating RD‐specific into medical curricula, promoting continuous professional development through specialized training events, and enhancing the visibility of reliable RD information sources. These measures are crucial for improving early diagnosis and management of RDs, ultimately enhancing patient care and outcomes in Türkiye.

## 1 Introduction

Rare diseases (RDs), characterized by their infrequent occurence within the general population, pose substantial diagnostic and therapeutic challenges due to their low prevalence and inherent complexity. Although a universally accepted definition is lacking, each country or region defines RDs within the framework of its unique cultural, demographic, resource‐based and and healthcare contexts [1]. The European Union (EU), through the Community Action Program for RDs from 1999 to 2003, defined RDs as conditions affecting fewer than 5 in 10,000 individuals [2]. Türkiye, though lacking an official definition for RDs, generally adheres to this EU benchmark [3].

According to Orphanet, 71.9% of RDs have a genetic origin and 69.9% manifest during childhood [4, 5]. Currently, over 10,000 rare and genetic diseases are recognized, with approximately 250 new rare diseases described annually. Despite progress in understanding and diagnosis technology, around 95% of RDs still remain without a curative treatment, and half of the affected population comprises children [6]. Diagnosis delays, often referred to as the “Diagnostic Odyssey,” are common and result in misdiagnoses and prolonged uncertainty [7].

In Türkiye, RDs are notably prevalent due to high rates of consanguinity, especially in rural and socio‐economically disadvantaged regions. This elevates the incidence of autosomal recessive disorders such as Spinal Muscular Atrophy (SMA), Familial Mediterranean Fever (FMF), and Beta‐Thalassemia. While the national newborn screening program includes some of these conditions, many remain undetected, especially those with variable or late‐onset presentations. Diagnostic limitations are further compounded by uneven access to genetic testing and a shortage of trained specialists [8, 9, 10, 11, 12, 13, 14, 15, 16, 17]. Table S1 summarizes ten rare genetic diseases frequently encountered in Türkiye, including their estimated prevalence and genetic basis.

One major barrier to timely diagnosis is inadequate awareness among healthcare professionals [18]. Insufficient inclusion of RDs in medical education, coupled with limited research and clinical literature, complicates accurate diagnosis. Across Europe, about 30 million people live with RDs, nearly half of whom remain undiagnosed [19]. Studies indicate that 25% of RD patients wait between 5 and 30 years for an accurate diagnosis, and 40% receive at least one misdiagnosis during that time [20]. Similarly, the 2013 Rare Disease Impact Report revealed that patients in the United Kingdom consulted an average of eight physicians and received two or three incorrect diagnoses before obtaining the correct diagnosis [21].

Medical education often prioritizes common conditions, leading many physicians to underestimate the likelihood of encountering RDs in practice [22]. In fact, a study in Australia found that approximately 8% of the population lives with a known rare disease, a prevalence comparable to that of diabetes or asthma [23]. In response, the EU Council in 2009 recommended improved training for health professionals to enhance awareness and diagnostic capacity for RDs [2]. Similarly, Türkiye's 2023–2027 RDs Health Strategy Document and Action Plan [24] emphasizes the need to assess and enhance RD‐related training for healthcare professionals. Countries such as Belgium [18], Spain [25] and Poland [26] have already begun addressing this through curriculum reform and needs assessments. However, in Türkiye, there remains a gap in understanding the educational needs of healthcare professionals regarding RDs.

This cross‐sectional study aimed to evaluate RD awareness, knowledge, and educational needs among physicians and senior medical students in Türkiye. It seeks to identify deficiencies in training and information access while exploring preferred learning modalities. These findings are intended to inform strategies that improve RD education and ultimately contribute to better diagnostic and treatment outcomes.

## 2 Materials and Methods

### 2.1 Study Population and Sample Size Determination

This study included a diverse cohort of healthcare professionals and advanced medical students, to enable a comprehensive analysis of RD awareness across various levels of medical training and specialties. The participants consisted of pediatric specialists from various subspecialties, such as general pediatrics, pediatric neurology, pediatric endocrinology, pediatric metabolism and pediatric hematology/oncology. Moreover, physicians from non‐pediatric specialities were included to provide a basis for comparing awareness and knowledge across different medical disciplines. The study further incorporated 5th and 6th‐year medical students from three prominent medical faculties in Türkiye: Atatürk University (A University), Başkent University (B University), and Istanbul University (I University). These students, nearing to transition to clinical practice, represent a critical group for assessing future readiness of physicians to manage the challenges related to RDs.

A total of 398 valid responses (response rate: 79.6%) were collected and included in the final analysis. Only eight responses were excluded from the analysis due to discrepancies that rendered them unrepresentative of the targeted population. A priori power analysis determined a minimum required sample size of 210 participants, assuming an effect size of 0.72 (Cohen's d), *α* = 0.05, and 80% power. The final sample size exceeded this threshold, ensuring sufficient statistical power. Participants were recruited through emails and online platforms, ensuring broad representation across specialties and training levels. Ethical approvals were obtained from the respective universities before the study's commencement (Approval numbers: E‐42190979‐000‐2300163716, E‐62310886‐300‐223065, E‐59763478‐302.99‐2203049). Participation was voluntary and anonymous, with no personal data collected.

During the data collection process, survey forms were disseminated to physicians and medical students via email and online platforms. Participants were assured of anonimity, with no personal data collected. This qualitative study aimed to assess the awareness levels, informational needs, and information acquisition preferences of physicians and medical students concerning RDs.

The study addressed the following research questions:

1. How do awareness levels and information acquisition methods regarding RDs differ between physicians and medical students?
2. What differences exist in knowledge levels about RDs between pediatric specialists and non‐pediatric physicians?
3. How are information acquisition processes regarding RDs shaped and structured for physicians and medical students?

### 2.2 Data Collection Tool

The data collection tool was developed by researchers based on methodologies from prior studies [18, 26, 27]. The survey included six sections: 1. *Personal Information*, gathered demographics data including gender, age, educational institution, year of study, and medical specialty. 2. *Knowledge Assessment* assessed respondents' knowledge about RDs. 3. *Awareness Evaluation evaluated* participants' awareness of RD‐related topics. 4. *The Education Review* reviewed the education received during medical training concerning RDs, 5. *Information Needs and Learning Modalities*, examined the participants' informational needs and preferred methods for acquiring information about RDs. 6. *Policy Awareness* evaluated participants' knowledge with national RD policies in Türkiye.

### 2.3 Medical Curriculum Analysis

To evaluate the extent and depth of RD content in medical training, curricula from A University, B University, and I University were reviewed. Course syllabi, annual schedules, and documentation pertaining to RD‐related education were analyzed. Instructional hours dedicated explicitly to RDs were calculated for each year, providing insights into the depth and integration of RD education. The analysis aimed to measure and to identify disparities in training coverage and to evaluate how well RDs are incorporated into different specialties, including internal medicine, pediatrics, obstetrics and gynecology, otolaryngology, ophthalmology, clinical genetics, urology, general surgery, physical therapy, orthopedics, dermatology, public health, plastic surgery, infectious diseases, and pediatric surgery. Databases such as Orphanet and the National Organization for Rare Disorders (NORD) were used to align curricula with recognized RD classifications.

### 2.4 Data Analysis

The statistical data analysis was conducted using SPSS version 28.0. Descriptive statistics were presented as frequencies and percentages. Chi‐square tests were utilized to anlayse independent categorical data. When chi‐square tests were unmet, the Fischer's exact test was applied. Curriculum analysis supplemented quantitative findings by evaluating the number of instructional hours and integration of RD content across disciplines.

This multi‐method approach ensured a robust analysis of knowledge, awareness, and education gaps regarding rare diseases among physicians and medical students in Türkiye.

## 3 Results

### 3.1 Participant Demographics

A total of 398 individuals participated in the study, comprising 258 physicians and 140 medical students. Among the physicians, 144 (55.8%) were female and 114 (44.2%) were male. In terms of specialty, 100 were pediatric specialists (including general pediatrics, pediatric neurology, endocrinology, metabolism and hematology/oncology), while 158 were enrolled from non‐pediatric specialties. Medical students were enrolled from A University, B University, and I University. Detailed demographic information is presented in Tables 1 and 2.

**Table 1:** Socio‐demographic characteristics of physicians participating in the study.

		*n*	%
Gender	Female	144	55.8
	Male	114	44.2
Age (yrs)	< 30	61	23.6
	30–45	120	46.5
	45–60	73	28.3
	≥ 60	4	1.6
Pediatric specialist Non‐pediatric specialist		100	38.7
		158	61.2

Abbreviations: %, percentage; n, number; yrs, years.

**Table 2:** Socio‐demographic characteristics of medical students participating in the study.

		A University		B University		I University	
		*n*	%	*n*	%	*n*	%
Age (yrs)	< 30	48	100	41	100	43	100
Gender	Female	19	39.6	22	53.7	12	27.9
	Male	29	60.4	19	46.3	31	72.1
Year of Study	5th Year	23	47.9	19	46.3	28	65.1
	6th Year	25	52.1	22	53.7	15	34.9

Abbreviations: A University, Atatürk University; B University, Başkent University; I University, Istanbul University; %, percentage; n, number; yrs, years.

### 3.2 Knowledge of Rare Diseases

Pediatric specialists rated their knowledge of RDs significantly higher than non‐pediatric specialists. Specifically, 83% of pediatric specialists rated their knowledge as ‘3’ or above, compared to 56.3% of non‐pediatric specialists (*p* < 0.05). This indicates a statistically significant difference in self‐assessed knowledge between the two groups (Table 3).

**Table 3:** Self‐reported knowledge levels of rare diseases among physicians.

		Non‐pediatric specialists		Pediatric specialists		*p*	
		*n*	%	*n*	%		
Knowledge level of rare diseases	1 (very low)	15	9.5	1	1.0	***0.001***	**χ** ^2^
	2 (low)	54	34.2	16	16.0		
	3 (moderate)	66	41.8	45	45.0		
	4 (high)	21	13.3	31	31.0		
	5 (very high)	2	1.3	7	7.0		

*Note:* Bold values are statistically significant.

Abbreviations: %, percentage; n, number; χ^2^, chi‐square (Fischer test).

Conversely, among students, no significant differences in self‐assessed knowledge of RDs was observed, with the majority rating their knowledge as low to very low (*p* > 0.05) (Table 4).

**Table 4:** Self‐reported knowledge levels of rare diseases among medical students.

		A University		B University		I University			
		*n*	%	*n*	%	*n*	%	*p*	
Knowledge level of rare diseases	1 (very low)	4	8.3	6	14.6	9	20.9	0.38	**χ** ^2^
	2 (low)	24	50.0	23	56.1	21	48.8		
	3 (moderate)	18	37.5	11	26.8	12	27.9		
	4 (high)	2	4.2	1	2.4	1	2.3		

Abbreviations: A University, Atatürk University; B University, Baskent University; I University, Istanbul University; %, percentage; n, number; χ^2^, chi‐square.

### 3.3 Awareness Levels Among Physicians

Pediatric specialists predominantly rated their awareness of RDs as ‘3’ (42%, *n* = 42) and ‘4’ (26%, *n* = 26). In contrast, non‐pediatric specialties most frequently rated their awareness as ‘2’ (39.9%, *n* = 63) and ‘3’ (37.3%, *n* = 59). Pediatric specialists rated their RD knowledge significantly higher than non‐pediatric specialists (*p* < 0.05) (Table 5). Regarding diagnostic timelines, 56% of pediatric specialists estimated that diagnosis of an RD typically takes more than 3 years, while 25% estimated it to be between 1 and 3 years. In comparison, 34.8% of non‐pediatric specialists estimated the diagnosis time to be over 3 years, and 25.9% estimated it to be between 1 and 3 years. Despite an estimated national prevalence of 5 to 6.5 million individuals affected by RDs in Türkiye [3], 78.5% of non‐pediatric specialists and 85% of pediatric specialists underestimated the affected population, believing it to be fewer than one million.

**Table 5:** Physicians self‐reported level of rare diseasesRDs awareness.

		Non‐pediatric specialists		Pediatric specialists		*p*	
		*n*	%	*n*	%		
Awareness level of rare diseases	1 (very low)	15	9.5	3	3.0	***0.001***	**χ** ^2^
	2 (low)	63	39.9	20	20.0		
	3 (moderate)	59	37.3	42	42.0		
	4 (high)	18	11.4	26	26.0		
	5 (very high)	3	1.9	9	9.0		

*Note:* Bold values are statistically significant.

Abbreviations: %, percentage; n, number; χ^2^, chi‐square (Fischer test).

### 3.4 Evaluation of Medical Education

When asked about the adequacy of time allocated to RDs in medical education, only 7% of pediatric specialists and 3.8% of non‐pediatric specialists considered it sufficient, while 18% and 24.1%, respectively, disagreed with this assessment (Table 6). Notably, all pediatric specialists agreed that the time spent on RDs is important, whereas 7.6% of non‐pediatric specialists believed it might be excessive. Among medical students, 2.1% from A University, 7.3% from B University, and 7% from I University felt their training on RDs was adequate. Conversely, 35.4%, 46.3%, and 32.6% from these institutions, respectively, found it insufficient and 29.2%, 22%, and 16.3% expressed a need for additional time on this topic.

**Table 6:** Physician evaluation of time allocated to rare diseases in medical education.

	Non‐pediatric specialists		Pediatric specialists	
	*n*	%	*n*	%
**During your medical (specialization) training, was a significant amount of time allocated to the study of rare diseases?**				
**Yes**	6	3.8	7	7.0
**Yes,** but it would have been beneficial to allocate additional time	35	22.2	20	20.0
**Yes,** it is necessary	16	10.1	21	21.0
**Yes,** quite extensively	11	7.0	6	6.0
**No**	38	24.1	18	18.0
**No,** it is not necessary	12	7.6	—	—
**No,** but the time allocated to it was quite extensive	5	3.2	—	—
**No,** but additional time should have been allocated to it	35	22.2	28	28.0

Abbreviations: %: percentage; n, number.

### 3.5 Perceived Usefulness of Education

Regarding training usefulness, 31% of pediatric specialists rated it as ‘very useful—useful’ compared to 19% of non‐pediatric specialists. The perceived usefulness differed significantly between the two groups (*p* < 0.05) (Table 7).

**Table 7:** Evaluation of the usefulness of medical education on rare diseases by physicians.

	Non‐pediatric specialists		Pediatric specialists			
	*n*	%	*n*	%	*p*	
**How effective and practically applicable is the medical education you received as a specialist for the diagnosis of rare diseases?“**						
**Very useful.** I feel confident in my ability to diagnose rare diseases.	4	2.5	8	8.0	***0.042***	**χ** ^2^
**Useful.** I have sufficient knowledge and practice to make a diagnosis.	26	16.5	23	23.0	0.192	**χ** ^2^
**Average.** My medical education did not prepare me perfectly.	72	45.6	45	45.0	0.929	**χ** ^2^
**Not useful**. I feel my medical education did not prepare me for this.	9	5.7	7	7.0	0.672	**χ** ^2^
**Insufficient**. I prefer to refer the patient to another specialist for diagnosis.	47	29.7	17	17.0	***0.021***	**χ** ^2^

Abbreviations: %, percentage; n, number; χ^2^, chi‐square (Fischer test).

### 3.6 Familiarity With Information Sources

Regarding the necessity of information on RDs in their daily practice, 35% of pediatric specialists and 26.6% non‐pediatric specialties reported require such information. Conversely, 7% of pediatric specialists and 7.6% of non‐pediatric specialties indicated that they do not need to require this information in their routine practice. Additionally, 14% of pediatric specialists and 24.1% of non‐pediatric specialists stated that they would only prioritize RDs when encountering patients diagnosed with such conditions. No statistically significant difference was observed between pediatric specialists and non‐pediatric specialties in this context (Table 8).

**Table 8:** Information needs regarding rare diseases in daily clinical practice among physicians.

	Non‐pediatric specialists		Pediatric specialists		
	*n*	%	*n*	%	*p*	
**Do you require information about rare diseases for your daily pratice?**						
**Yes**	42	26.6	35	35.0		
**Yes**, but I am unsure where to find reliable information	17	10.8	8	8.0		
**Yes,** because I seek to gain more knowledge	31	19.6	29	29.0		
I only require such information when treating a patient with a rare disease	38	24.1	14	14.0	0.199	**χ** ^2^
**No**, because I am not interested in information about rare diseases	8	5.1	3	3.0		
**No,** I don't have the time to read them	10	6.3	4	4.0		
**No**	12	7.6	7	7.0		

Abbreviations: %, percentage; n, number; χ^2^, chi‐square.

### 3.7 Preferred Learning Modalities

A significant portion of pediatric specialists (18%) have undergone re‐education on RDs, with 60% expressing interest in further training. In contrast, only 8.9% of non‐pediatric specialists have pursued re‐education, although 44.9% indicated interest. Among medical students, 32.6% expressed a desire to learn more about RDs, while 29.5% prefered courses focused on more common conditions. These results underscore the importance of prioritizing RDs within medical education.

Additionally, 36.4% of physicians and 78% of medical students were unfamiliar with major information sources on rare diseases. Pediatric specialists were significantly more familiar with sources like Orphanet and Eurordis compared to non‐pediatric specialists (*p* < 0.05). These results highlight the importance of improving access to and awareness of rare disease resources for both physicians and medical students. When asked about their most needed information on RDs, pediatric specialists identified the most needed information on RDs diagnostic tests (51%), screening programs (49%), clinical cases (49%), orphan drugs (47%), and symptomatology (36%). Non‐pediatric specialists prioritized screening programs (48.1%), diagnostic tests (43%), symptoms (38.6%), clinical cases (34.8%), and basic and translational research (34.8%) as top needs. Additionally, 8% of pediatric specialists and 8.9% of non‐pediatric specialists stated that they only need information on treatable RDs.

Regarding preferred learning channels, both groups favored conferences and symposiums (70% of pediatric specialists and 51.9% of non‐pediatric specialists), followed by consultation with medical experts and healthcare centers (46% and 46.2%, respectively), specialty associations (41% and 39.2%, respectively), and periodicals or journals (33% and 32.3%, respectively). Remote education and webinars were also favored by a smaller percentage (33% and 30.4%, respectively). Medical students primarily preferred acquiring information from medical experts or healthcare centers (59.1%), conferences and symposiums (46.2%), and personalized training with specialized physicians during early education (42.4%) (Table 9).

**Table 9:** Preferred sources of information on rare diseases among physicians and medical students.

	Non‐pediatric specialists		Pediatric specialists		Medical students	
	*n*	%	*n*	%	*n*	%
**From which sources do you prefer to obtain information about rare diseases?**						
Medical experts/healthcare centers	73	46.2	46	46.0	78	59.1
Conferences, congresses, symposiums	82	51.9	70	70.0	61	46.2
The early years of specialist training	24	15.2	24	24.0	56	42.4
Medical specialty associations	62	39.2	41	41.0	55	41.7
The early years of medical education	15	9.5	17	17.0	49	37.1
Periodicals, journals, newspapers, advertisements	51	32.3	33	33.0	42	31.8
Distance Learning/webinars	48	30.4	33	33.0	35	26.5
Continuing education and re‐training	33	20.9	27	27.0	28	21.2
Public authorities or agencies	27	17.1	16	16.0	20	15.2
Patient organizations	11	7.0	10	10.0	16	12.1
Videos and films	17	10.8	11	11.0	15	11.4
Patients	9	5.7	5	5.0	14	10.6
E‐mails	14	8.9	7	7.0	13	9.8
Brochures, leaflets, information booklets	19	12.0	19	19.0	12	9.1
Orphan drug manufacturers	6	3.8	14	14.0	7	5.3
Advertising campaigns	6	3.8	4	4.0	4	3.0
I do not need information about rare diseases	4	2.5	0	0.0	9	6.8

Abbreviations: %, percentage; n, number.

In the final section of the study, participants were questioned about Türkiye's policies on RDs. It was revealed that 66.7% of physicians and 87.9% of medical students were unaware of a national rare disease program in Türkiye. Furthermore, 58.9% of physicians and 69.7% of medical students were uncertain if records of individuals with RDs are centrally maintained, and 41.5% of physicians and 64.4% of medical students were unsure about the reimbursement status of orphan drugs in Türkiye.

### 3.8 Analysis of Curriculum Allocation for Rare Diseases

An analysis of instructional hours dedicated to RDs within the curricula of the three universities involved in this study, revealed that formal instruction typically begins in the third year of medical education. At A University, the distribution of instructional hours allocated to RDs across the medical curriculum is as follows: 60 h in the third year, 99 h in the fourth year, 76 h in the fifth year, and 61.5 h in the sixth year, totaling 296.5 h. At B University, the allocation includes 132 h in the third year, 52.4 h in the fourth year, and 53.3 h in the fifth year, resulting in a cumulative total of 237.7 h. At I University, the distribution comprises 35.9 h in the third year, 88 h in the fourth year, and 52 h in the fifth year, totaling 175.9 h (Table 10).

**Table 10:** Allocation of instructional time for rare diseases in medical curricula by universities.

	3rd year of medical school	4th year of medical school	5th year of medical school	6th year of medical school	Total time devoted on rare diseases
A University	60 h	99 h	76 h	61.5 h	296.5 h
B University	160 h	52.5 h	51.6 h	—	264.7 h
I University	35.9 h	88 h	52 h	—	175.9 h

Abbreviation: hrs, hours.

Beyond quantifying instructional hours, a qualitative review of course content indicates that rare disease education is primarily embedded within lectures in medical genetics, pediatrics, clinical biochemistry, and neurology. For example, A University incorporates case‐based learning modules in pediatrics and metabolic diseases, emphasizing diagnostic reasoning and clinical decision‐making in rare cases. B University offers structured modules on genetic counseling and diagnostic pathways for metabolic disorders and incorporates simulation‐based exercises such as enzyme assay interpretation and newborn screening protocols. I University addresses rare diseases during pediatric and internal medicine clerkships, often through supplementary seminars or guest lectures delivered by subspecialists. However, across all three universities, there is limited formal assessment of students' competencies specifically related to rare disease recognition and management of rare diseases, and practical exposure to such conditions remains sporadic. This gap between theoretical instruction and hands‐on clinical experience may contribute to the consistently low‐self‐assessed knowledge ratings reported by students, regardless of the differences in curricular structure.

## 4 Discussion

### 4.1 Knowledge of Rare Diseases

A significant proportion of medical students, 58.3% at A University, 70.7% at B University, and 69.8% at I University, rated their knowledge of RDs as ‘Poor’ or ‘Very Poor,’ despite varying hours dedicated to rare disease education across these institutions (A University: 296.5, B University: 264.7, I University: 175.9 h). However, no significant difference was observed in self‐assessed knowledge across institutions. This finding aligns with prior research nemphasizing the limited effectiveness of current curricula in fostering RD knowledge and supporting the call for structured, dedicated instruction rather than fragmented coverage under genetics topics [27, 28, 29, 30].

Our curriculum analysis confirms that greater instructional time alone does not correlate with improved perceived competence. This suggests that pedagogical quality, integration with clinical scenarios, and active learning methods may be more impactful. Even students from institutions with the most RD‐focused hours did not report proportionally higher confidence. Furthermore, limited clinical exposure and the absence of formal assessments on RD topics likely contribute to the disconnect between instruction and preparedness. Notably, 53% of students rated their education on RDs as ‘Insufficient,’ yet 18.9% were uninterested in further learning, and 29.5% preferred focusing on more common diseases, highlighting the need for content that is not only informative but also engaging and clinically relevant.

For practicing physicians, pediatric specialists generally rated their knowledge as ‘good’ or ‘average,’ whereas non‐pediatric specialists tended toward ‘average’ or ‘poor.’ This disparity in knowledge levels is consistent with other studies, which have shown that pediatric specialists tend to have better knowledge of RDs than other physicians, although still not at the level of pediatric subspecialists [18]. The global trend of insufficient knowledge among physicians about RDs (e.g., 94.6% in Poland, 94.7% in China) suggests that current medical curricula worldwide, including in Türkiye, are inadequate in preparing future healthcare professionals to address this significant public health issue [26, 31].

### 4.2 Curriculum Content Integration and Learning Outcomes: A Comparative Summary

To meaningfully interpret RD curriculum design, we compared instructional hours across three universities with students' self‐assessed knowledge levels. To meaningfully interpret RD curriculum design, we compared instructional hours across three universities with students' self‐assessed knowledge levels. Notably, despite differences in curricular exposure ranging from early integration to clinical‐year emphasis, no significant correlation was observed between hours of instruction and perceived knowledge. This finding aligns with prior literature emphasizing that instructional quantity alone does not guarantee competence in rare disease education [32].

Our comparative analysis revealed distinct models: A University adopts a longitudinal, case‐based approach; B University emphasizes early foundational content supported by simulation; I University integrates RD content into clinical clerkships and seminars. Despite these structural differences, all lacked structured clinical exposure and outcome‐based assessments tailored to RDs, an omission widely regarded as essential for effective medical training [32, 33].

This study is distinguished by its integrated analysis of both curricular design and student‐reported outcomes, revealing a disconnect between the extent of educational investment and students' self‐assessed confidence. While prior studies have identified general curricular gaps, our findings emphasize the need not merely for additional content, but strategically integrated, experience‐driven instruction that effectively bridges theory and clinical practice.

### 4.3 Awareness Levels Among Medical Professionals

There is a significant difference in awareness levels between pediatric specialists and non‐pediatric physicians regarding RDs, with pediatric specialists generally reporting higher awareness (*p* < 0.001). Interestingly, while 49.4% of non‐pediatric specialists rated their awareness as ‘poor,’ 59.5% reported having suspected RDs in their patient's multiple times, compared to 71% among pediatric specialists. This discrepancy suggests that while awareness may vary by specialty, it does not necessarily reflect the frequency with which RDs are encountered in practice. The finding that many non‐pediatric physicians, particularly general practitioners, suspect RDs despite low self‐reported awareness is significant. Early diagnosis is crucial for RDs patients, and improving awareness among all physicians, especially those who are first points of contact, is essential for better patient outcomes [33] (Figure S1).

Quality of life can be severely affected due to delayed diagnosis, contributing to various psychiatric and developmental problems [34]. The findings of this study reveal that both physicians and medical students have low awareness of RDs, with 87.6% of physicians and 98.5% of students unaware of RDs prevalence in Türkiye, and more than half unable to estimate the average time to diagnosis. The results emphasize the urgent need for enhanced education to improve healthcare professionals' ability to recognize and manage RDs, ultimately strengthening their role in addressing these conditions.

### 4.4 Information Needs and Resource Gaps

Critical knowledge gaps among pediatric specialists highlight the complexities associated with the diagnosis and management of RDs, with a particular emphasis on the diagnostic tests (51%), the screening programs (49%), and the clinical case studies (49%). The priority points to an urgent need for resources that can improve both diagnosis precision and management techniques. Similarly, non‐pediatric specialists prioritized knowledge regarding screening programs (48.1%) and diagnostic tests (43%), reflecting the broad spectrum of health concerns they manage and underscoring the importance of early detection and timely intervention. The consistent conclusion across multiple studies that healthcare professionals encounter significant knowledge gaps in managing RDs further emphasizes the urgency of addressing these gaps through targeted educational initiatives [35, 36]. Providing comprehensive education on RDs to both medical students and practicing physicians receive is crucial for developing a more informed and competent clinical practice, ultimately enhancing patient care and improving the quality of life for those affected by RDs.

The data analysis highlighted a significant lack of awareness among medical students regarding rare disease information sources, with 78% unable to identify any of the available resources. By contrast, pediatric specialists and non‐pediatric physicians most commonly cited Orphanet, Eurordis, and NORD as key sources of reliable RDs information, emphasizing their importance in delivering reliable and most updated resources. Employing digital tools, like those suggested in Belgium for correlating symptoms with potential diagnoses and treatment options, could significantly contribute to bridging these knowledge gaps and enhancing healthcare professionals' capabilities in managing RDs [18].

### 4.5 Evaluation of Medical Education

Most physicians considered their RD education inadequate, with 45.3% assessing it as ‘average’ in terms of usefulness. Despite 24% feeling insufficiently prepared to diagnose RDs, and often choosing to refer patients to specialists, pediatric specialists reported greater educational advantages. Enhancing RD education during medical school and through continuous professional development is essential. About 74.2% of students felt insufficient time was allocated to RDs, underscoring the need for updated curricula and additional training.

### 4.6 Curriculum Allocation for Rare Diseases

The review of curriculum allocation for RDs across A University, B University, and I University reveals significant variability in the instructional time dedicated to this critical aspect of medical education. A University allocated 296.5 h for rare disease education. This curriculum is well‐balanced, enhancing both theoretical and practical skills while B University dedicated 237.7 h, primarily focusing heavily in the third year, to establish foundational knowledge early; in contrast, I University provides 175.9 h, focusing on integrating rare disease education in the fourth year to emphasize practical application. These varying approaches to rare disease education reflect each institution's prioritization and may impact medical training outcomes. The differences in instructional hours could be influenced by geographical context, the regional prevalence of specific RDs, and whether the institution is public or private, highlighting the role of institutional priorities, resource allocations, and local healthcare demans in shaping curriculum design. A University, a public institution in Erzurum (eastern Türkiye), may prioritize RDs prevalent in its region, while B University, a private university in Ankara (central Türkiye), may focus to health needs of central Türkiye, and I University, a public university, in western Türkiye, likely addresses on RDs more common in its locality. This geographical diversity highlights how regional disease patterns influence medical curricula and educational priorities. Additionally, the status of a university–public or private–might also shape its curriculum: B University teaches RDs intensively early in medical education, while A University and I University integrate rare disease education comprehensively throughout their entire curricula, with A University fostering well‐rounded expertise, while B University establishing foundational knowledge reinforced by practical experience, and I University emphasizing clinical application in the fourth year. These variations suggest that the timing and integration of rare disease education are vital for effective medical training, indicating that a balanced approach could enhance RDs education for future medical professionals and warranting further exploration of these educational strategies outcomes. Future curriculum development should prioritize not only increasing the instructional time for RDs but also ensuring integration with clinical experiences, use of diagnostic case simulations, and formal competency assessments to enhance preparedness and confidence among future physicians.

### 4.7 Broader Implications for Curriculum Design

These findings underscore the importance of designing RD education that is not only quantitatively sufficient in hours but also pedagogically structured for impact. Medical curricula should adopt a hybrid model that includes vertical integration across academic years, active learning strategies such as case‐based discussions and simulations, and direct patient encounters where feasible. Institutions should also consider incorporating RD content into existing modules, such as pediatrics, internal medicine, and clinical genetics while offering elective opportunities for deeper engagement. Standardized outcome‐based learning objectives related to RDs should be included in national medical education frameworks to ensure consistency and accountability. Such reforms can bridge the gap between knowledge and competency, helping to ensure that graduates are not only informed about RDs but are also capable of identifying and managing them in practice.

In alignment with earlier findings, our study found that medical students generally lack knowledge and awareness of RDs, with a consensus on the need for improved undergraduate training. Early studies across different countries indicate a persistent gap in understanding RDs [26, 29, 30, 37]. Though direct comparisons are challenging due to differing methodologies, many studies reveal low understanding of RDs and their public health implications, particularly in primary prevention and the importance of premarital genetic counseling [38, 39, 40].

In several studies, medical students have recognized the need for improved RDs education, advocating for elective courses, expert lectures, and research involvement. The analysis of our work identified significant knowledge gaps among medical students particularly regarding RDs and orphan drugs, with no observable improvement across different years of study. Those gaps in undergraduate education can be effectively addressed through extracurricular initiatives that pair medical students with experienced healthcare providers and RD patients. Ultimately, these timely diagnosis and treatment by deepening students' understanding of RDs and enhabcing medical training [41, 42, 43].

### 4.8 Rare Disease Policy Awareness and Implications

Many healthcare professionals surveyed lacked awareness of key aspects of Türkiye's rare disease policies, such as a national program, central registry, and orphan drugs reimbursement which hinders effective healthcare delivery and underscores the need for better education and communication. Enhancing knowledge of these policies is essential for promoting early diagnosis and appropriate management of rare diseases, and strengthening policies like a centralized registry and clear orphan frug reimbursement could significantly improve the quality of life for affected ones.

### 4.9 Limitations

The findings of this study are influenced by the demographic profile of the participating physicians, with 46.5% falling within the age range of 30 to 45 years and only 1.6% aged 60 years or older. This disproportionate representation of younger physicians may limit the applicability of the findings to the broader physician population. Future research should address these limitations by incorporating a more diverse physician group, including a wider age range. Such improvements would enhance the accuracy and applicability of findings, enabling the development of more effective strategies to advance RDs education and knowledge among healthcare professionals.

## 5 Conclusion

Our findings underscored a critical gap in the competence of healthcare professionals in diagnosing and managing RDs. The study revealed a substantial disparity in basic knowledge between pediatric specialists and non‐pediatric specialists, with no significant differences in self‐assessed knowledge among medical students from universities with varying curricula on RDs. Despite expressing interest in further education on RDs, participants displayed considerable gaps in awareness of available information sources. This lack of awareness significantly hampers efforts to improve rare disease knowledge among healthcare professionals. To address these deficiencies and issues, we propose: **1. Curricular Enhancement**: Increase RD instructional time and enrich curricula with clinical case studies covering diagnosis, diagnostic tests, screening programs, and prevention. **2. Ongoing Education**: Facilitate continuous learning opportunities through congresses, symposiums, and conferences to support and update the knowledge of both current practitioners and medical students. **3. Improved Access to Information**: Enhance the visibility and accessibility of reliable information sources related to RDs to ensure healthcare professionals are well‐informed in accessing information.

Implementing these recommendations will bridge the existing knowledge gaps, foster earlier and more accurate diagnosis, and improve RD management, ultimately enhancing the quality of care for patients with RDs and contribute to more effective healthcare outcomes.

## Disclosure

The authors alone are responsible for the content and writing of the article.

## Conflicts of Interest

The authors declare no conflicts of interest.

## Supporting information


**Figure S1:** Diagnostic algorithm for approaching a suspected rare disease case in clinical practice.
**Table S1:** Overview of Ten Rare Genetic Diseases Commonly Observed in Türkiye, Including Estimated Prevalence, Typical Age at Diagnosis, and Genetic Mechanisms.

## Data Availability

The data that support the findings of this study are available from the corresponding author upon reasonable request.

## References

[1] Y. Çolak , “Nadir Hastalıklar Epidemiyolojisi,” in Tüm Yönleriyle Nadir Hastalıklar, ed. Ö İnce and M. D. Pak (Nobel Akademik Yayıncılık, 2019), 15–25.

[2] Council of the European Union , Council Recommendation of 8 June 2009 on an Action in the Field of Rare Diseases, 2009/C 151/02, https://eur‐lex.europa.eu/LexUriServ/LexUriServ.do?uri=OJ:C:2009:151:0007:0010:EN:PDF.

[3] Türkiye Büyük Millet Meclisi (TBMM) , ALS, SMA, DMD, MS Hastalıklarında Ve Kesin Tedavisi Bilinmeyen Diğer Hastalıklarda Uygulanan Tedavi ve Bakım Yöntemleri İle Bu Hastalıklara Sahip Kişiler Ve Yakınlarının Yaşadıkları Sorunların Ve Çözümlerinin Belirlenmesi Amacıyla Kurulan Meclis Araştırması Komisyonu İstanbul Toplantı Tutanakları (TBMM Yayınları, 2019).

[4] Orphanet , *About Rare Diseases*, accessed June, 5, 2025, https://www.orpha.net/.

[5] S. Nguengang Wakap , D. M. Lambert , A. Olry , et al., “Estimating Cumulative Point Prevalence of Rare Diseases: Analysis of the Orphanet Database,” European Journal of Human Genetics 28, no. 2 (2020): 165–173. 31527858 10.1038/s41431-019-0508-0 PMC6974615

[6] Global Genes , *Rare Disease Facts*, accessed June 5, 2025, https://globalgenes.org/rare-disease-facts/.

[7] A. Bauskis , C. Strange , C. Molster , and C. Fisher , “The Diagnostic Odyssey: Insights From Parents of Children Living With an Undiagnosed Condition,” Orphanet Journal of Rare Diseases 17, no. 1 (2022): 233. 35717227 10.1186/s13023-022-02358-x PMC9206122

[8] K. M. Celik , C. C. Kose , D. Kaya , K. Tekin , and F. Silan , “Spinal Muscular Atrophy Carrier Screening Program: Awareness and Attitude of Healthcare Professionals in Turkey,” Journal of Community Genetics 15, no. 6 (2024): 665–672. 39392569 10.1007/s12687-024-00737-4 PMC11645347

[9] Ö. Alparslan , B. H. Egeli , Y. Demirel , and S. Uğurlu , “The Prevalence of Familial Mediterranean Fever and Behçet's Disease: A Cross‐Sectional Study,” Archives of Rheumatology 35, no. 4 (2020): 609–613. 33758818 10.46497/ArchRheumatol.2020.7769 PMC7945712

[10] Y. Aydınok , Y. Oymak , B. Atabay , et al., “A National Registry of Thalassemia in Turkey: Demographic and Disease Characteristics of Patients, Achievements, and Challenges in Prevention,” Turkish Journal of Hematology 35, no. 1 (2018): 12–18. 28404539 10.4274/tjh.2017.0039 PMC5843769

[11] M. Esgi , H. Ergun , N. Y. Kaya , D. Y. Atakay , E. Erucar , and F. Celik , “Phenylketonuria From the Perspectives of Patients in Türkiye,” Orphanet Journal of Rare Diseases 19, no. 1 (2024): 78. 38378595 10.1186/s13023-024-03079-z PMC10880278

[12] K. Çıkı , C. Alavanda , E. I. Ceylan , T. Tanyalçın , and S. Kılavuz , “Comprehensive Analysis of Genotypic and Phenotypic Characteristics of Biotinidase Deficiency Patients in the Eastern Region of Türkiye,” Turkish Journal of Pediatrics 66, no. 5 (2024): 608–617. 39582447 10.24953/turkjpediatr.2024.5075

[13] D. Dogru , E. Çakır , T. Şişmanlar , et al., “Cystic Fibrosis in Turkey: First Data From the National Registry,” Pediatric Pulmonology 55, no. 2 (2020): 541–548. 31710166 10.1002/ppul.24561

[14] T. Güran , B. Tezel , M. Çakır , et al., “Neonatal Screening for Congenital Adrenal Hyperplasia in Turkey: Outcomes of Extended Pilot Study in 241,083 Infants,” Journal of Clinical Research in Pediatric Endocrinology 12, no. 3 (2020): 287–294. 32157855 10.4274/jcrpe.galenos.2020.2019.0182 PMC7499135

[15] E. Kurnaz , A. Türkyılmaz , O. Yaralı , A. S. Dönmez , and A. Çayır , “Genetic Analyses in a Cohort of Pediatric Patients With Congenital Hypothyroidism Based on Congenital Hypothyroidism Consensus Guideline,” Hormone Research in Paediatrics (2024): 1–15. 10.1159/000541898 PMC12795535 39378853

[16] B. Celik , S. C. Tomatsu , S. Tomatsu , and S. A. Khan , “Epidemiology of Mucopolysaccharidoses Update,” Diagnostics 11, no. 2 (2021): 273. 33578874 10.3390/diagnostics11020273 PMC7916572

[17] H. Dwedar , S. Al Dallal , M. Farghaly , et al., “EPH101 Prevalence of Sickle Cell Disease in the Middle East: A Systematic Literature Review,” Value in Health 27, no. 12 (2024): S240–S241.

[18] L. Vandeborne , E. van Overbeeke , M. Dooms , B. De Beleyr , and I. Huys , “Information Needs of Physicians Regarding the Diagnosis of Rare Diseases: A Questionnaire‐Based Study in Belgium,” Orphanet Journal of Rare Diseases 14, no. 1 (2019): 99. 31054581 10.1186/s13023-019-1075-8 PMC6500578

[19] European Commission , The Long Journey to a Rare Disease Diagnosis: Horizon Magazine. (2022), https://projects.research-and-innovation.ec.europa.eu/en/horizon-magazine/long-journey-rare-disease-diagnosis.

[20] EURORDIS , “The Voice of 12000 Patients,” in Experiences and Expectations of Rare Disease Patients on Diagnosis and Care in Europe. (EURORDIS, 2009), https://www.eurordis.org/publication/the-voice-of-12000-patients/.

[21] Shire, Rare Disease Impact Report: Insights From Patients and the Medical Community, (2013), https://globalgenes.org/wp-content/uploads/2013/04/ShireReport-1.pdf.

[22] P. Byrne , “Training Medical Students on Rare Disorders,” *Orphanet Journal of Rare Diseases 7*, no. Suppl 2 (2013): A15.

[23] E. J. Elliott , A. Nicoll , R. Lynn , V. Marchessault , R. Hirasing , and G. Ridley , “Rare Disease Surveillance: An International Perspective,” Paediatrics & Child Health 6, no. 5 (2001): 251–260. 20084246 PMC2804555

[24] T. C. Sağlık Bakanlığı , *Nadir Görülen Hastalıklar Eylem Planı 2023–2027*, (Sağlık Bakanlığı, 2023).

[25] E. Ramalle‐Gómara , E. Domínguez‐Garrido , M. Gómez‐Eguílaz , M. E. Marzo‐Sola , J. L. Ramón‐Trapero , and J. Gil‐de‐Gómez , “Education and Information Needs for Physicians About Rare Diseases in Spain,” Orphanet Journal of Rare Diseases 15, no. 1 (2020): 18. 31952528 10.1186/s13023-019-1285-0 PMC6969468

[26] D. Walkowiak and J. Domaradzki , “Are Rare Diseases Overlooked by Medical Education? Awareness of Rare Diseases Among Physicians in Poland: An Explanatory Study,” Orphanet Journal of Rare Diseases 16 (2021): 400. 34583737 10.1186/s13023-021-02023-9 PMC8479904

[27] D. Walkowiak , K. Bokayeva , A. Miraleyeva , and J. Domaradzki , “The Awareness of Rare Diseases Among Medical Students and Practicing Physicians in the Republic of Kazakhstan. An Exploratory Study,” Frontiers in Public Health 10 (2022): 872648. 35462837 10.3389/fpubh.2022.872648 PMC9031913

[28] E. Hristova‐Atanasova , G. Iskrov , I. Atanasov , A. Genc , and R. Stefanov , “What Is the Awareness of Rare Diseases Among Medical Students? A Survey in Bulgaria,” Orphanet Journal of Rare Diseases 18, no. 1 (2023): 213. 37491304 10.1186/s13023-023-02820-4 PMC10369688

[29] K. Jonas , M. Waligóra , M. Hołda , et al., “Knowledge of Rare Diseases Among Health Care Students–The Effect of Targeted Education,” Przeglad Epidemiologiczny 71, no. 1 (2017): 80–89. 28742309

[30] R. Jahanshahi , A. Nasirzadeh , M. Farzan , et al., “Iranian Future Healthcare Professionals' Knowledge and Opinions About Rare Diseases: Cross‐Sectional Study,” Orphanet Journal of Rare Diseases 17, no. 1 (2022): 366. 36175905 10.1186/s13023-022-02458-8 PMC9524105

[31] X. Li , X. Zhang , S. Zhang , et al., “Rare Disease Awareness and Perspectives of Physicians in China: A Questionnaire‐Based Study,” Orphanet Journal of Rare Diseases 16 (2021): 171. 33849615 10.1186/s13023-021-01788-3 PMC8042908

[32] R. M. Harden and J. M. Laidlaw , “Be Fair to Students: Four Principles That Lead to More Effective Learning,” Medical Teacher 35, no. 1 (2013): 27–31. 23121246 10.3109/0142159X.2012.732717

[33] S. Bavisetty , W. W. Grody , and S. Yazdani , “Emergence of Pediatric Rare Diseases: Review of Present Policies and Opportunities for Improvement,” Rare Diseases 1, no. 1 (2013): e23579. 25002987 10.4161/rdis.23579 PMC3932940

[34] J. Domaradzki and D. Walkowiak , “Medical Students' Knowledge and Opinions About Rare Diseases: A Case Study From Poland,” Intractable & Rare Diseases Research 8, no. 4 (2019): 252–259. 31890452 10.5582/irdr.2019.01099 PMC6929592

[35] T. Miteva , R. Jordanova , G. Iskrov , and R. Stefanov , “General Knowledge and Awareness on Rare Diseases Among General Practitioners in Bulgaria,” Georgian Medical News, no. 193 (2011): 16–19. 21617267

[36] Y. Zurynski , A. Gonzalez , M. Deverell , et al., “Rare Disease: A National Survey of Paediatricians' Experiences and Needs,” BMJ Paediatrics Open 1, no. 1 (2017): e000172. 29637168 10.1136/bmjpo-2017-000172 PMC5862166

[37] A. Flores , S. Burgos , and H. Abarca‐Barriga , “Knowledge Level of Medical Students and Physicians About Rare Diseases in Lima, Peru,” Intractable & Rare Diseases Research 11, no. 4 (2022): 180–188. 36457581 10.5582/irdr.2022.01079 PMC9709623

[38] M. Al‐Shafai , A. Al‐Romaihi , N. Al‐Hajri , N. Islam , and K. Adawi , “Knowledge and Perception of and Attitude Toward a Premarital Screening Program in Qatar: A Cross‐Sectional Study,” International Journal of Environmental Research and Public Health 19, no. 7 (2022): 4418. 35410099 10.3390/ijerph19074418 PMC8998822

[39] M. Eichholzer , O. Tönz , and R. Zimmermann , “Folic Acid: A Public‐Health Challenge,” Lancet 367, no. 9519 (2006): 1352–1361. 16631914 10.1016/S0140-6736(06)68582-6

[40] B. Medic , N. Divac , B. Stopic , et al., “The Attitudes of Medical Students Towards Rare Diseases: A Cross‐Sectional Study,” Vojnosanitetski Pregled 73, no. 8 (2016): 703–713. 29328568 10.2298/VSP150326094M

[41] V. Héon‐Klin , “European Reference Networks for Rare Diseases: What Is the Conceptual Framework?,” Orphanet Journal of Rare Diseases 12 (2017): 137. 28784158 10.1186/s13023-017-0676-3 PMC5547471

[42] A. Morgenthau , C. Margus , M. P. Mackley , and A. P. Miller , “Rare Disease Education Outside of the Classroom and Clinic: Evaluation of the RARE Compassion Program for Undergraduate Medical Students,” Genes 13, no. 10 (2022): 1707. 36292592 10.3390/genes13101707 PMC9601568

[43] C. Valcárcel‐Nazco , Y. Ramallo‐Fariña , R. Linertová , J. M. Ramos‐Goñi , L. García‐Pérez , and P. Serrano‐Aguilar , “Health‐Related Quality of Life and Perceived Burden of Informal Caregivers of Patients With Rare Diseases in Selected European Countries,” International Journal of Environmental Research and Public Health 19, no. 13 (2022): 8208. 35805867 10.3390/ijerph19138208 PMC9266302

