# Supplementary material for: Enhancing Rare Disease Awareness and Education Among Medical Professionals and Students in Türkiye
Source: J Eval Clin Pract. 2025 Aug 12;31(5):e70242. doi: 10.1111/jep.70242 (PMC12341651; doi:10.1111/jep.70242)
Supplement: Supplementary file 1 — Figure S1: Diagnostic algorithm for approaching a suspected rare disease case in clinical practice. Table S1: Overview of Ten Rare Genetic Diseases Commonly Observed in Türkiye, Including Estimated Prevalence, Typical Age at Diagnosis, and Genetic Mechanisms. [file JEP-31-0-s001.docx]

**Supplementary Table 1:** Overview of Ten Rare Genetic Diseases Commonly Observed in Türkiye, Including Estimated Prevalence, Typical Age at Diagnosis, and Genetic Mechanisms.

| **Disease** | **Prevalence (per 100,000)** | **Typical Age at Diagnosis** | **Genetic Mechanisms** |
| --- | --- | --- | --- |
| Spinal Muscular Atrophy (SMA) | 10-20 | Infancy | SMN1 gene deletion/mutations (8) |
| Familial Mediterranean Fever (FMF) | 100–200 | Childhood | MEFV mutations (AR)(9) |
| Beta- Thalassemia | ~2000 | Infancy/Childhood | HBB mutations (AR) (10) |
| Phenylketonuria (PKU) | 22.2 | Newborn | PAH mutations (AR) (11) |
| Biotinidase Deficiency (BTD) | 14.1 | Newborn | BTD gene mutations (AR) (12) |
| Cystic Fibrosis (CF) | 20–33 | Newborn | CFTR gene mutations (AR) (13) |
| Congenital Adrenal Hyperplasia (CAH) | ~6.7 | Newborn | CYP21A2 mutations (AR) (14) |
| Congenital Hypothyroidism (CH) | 25-29 | Newborn | TSHR, TPO, DUOX2 mutations (15) |
| Mucopolysaccaridosis III (MPSIII) | ~ >4.1 | Childhood | SGSH, NAGLU, HGSNAT, GNS mutations (AR) (16) |
| Sickle Cell Anemia | ~13.2 | Newborn | HBB gene mutations (AR)(17) |

For several conditions listed above, specific prevalence data in Türkiye are not publicly available. The figures provided are based on findings from recent disease-specific studies, as no centralized national registry or comprehensive public dataset currently exists (8-17).

**Supplementary Figure 1.** Diagnostic algorithm for approaching a suspected rare disease case in clinical practice

Patient presents with unexplained/multisystem symptoms

Detailed family and clinical history

Comprehensive physical examination

Basic laboratory and imaging investigations

If diagnosis remains unclear → consider rare disease

Consult databases (e.g., Orphanet, OMIM)

Order targeted genetic/metabolic testing

Refer to genetic/metabolic disease specialist

**Initiate diagnosis-specific management plan**

A step-by-step framework for evaluating patients with suspected rare diseases, integrating clinical suspicion, basic and specialized testing, referral, and use of rare disease resources such as Orphanet and expert consultation.
